# Clinical Efficacy and Safety of Massage for the Treatment of Restless Leg Syndrome in Hemodialysis Patients: A Meta-Analysis of 5 Randomized Controlled Trials

**DOI:** 10.3389/fpsyt.2022.843263

**Published:** 2022-04-11

**Authors:** Min Xia, Qien He, Guanghui Ying, Xiapei Fei, Wenjing Zhou, Xuelin He

**Affiliations:** ^1^Department of Nephrology, Beilun People’s Hospital, Ningbo, China; ^2^Kidney Disease Center, The First Affiliated Hospital, Zhejiang University School of Medicine, Hangzhou, China; ^3^Kidney Disease Immunology Laboratory, The Third Grade Laboratory, State Administration of Traditional Chinese Medicine of China, Hangzhou, China

**Keywords:** restless legs syndrome, hemodialysis, non-pharmacological interventions, massage, meta-analysis

## Abstract

**Aim:**

We conducted this meta-analysis to evaluate the clinical efficacy and safety of massage for the treatment of hemodialysis patients with restless leg syndrome (RLS).

**Methods:**

A comprehensive literature search was performed using the PubMed database, EMBASE database (*via* OVID), and the Cochrane Library in order to identify eligible randomized controlled trials (RCTs) published before August 31, 2021. After extracted essential data and assessed risk of bias of each eligible study, we calculated the pooled estimate of RLS score and safety after treatment. Statistical analysis was performed by using Review Manager 5.3.

**Results:**

Five studies involving 369 hemodialysis patients with RLS were analyzed. The RLS score after treatment [mean difference (MD), −12.01; 95% confidence interval (CI), −14.91 to −9.11] and mean difference of RLS score at the beginning and end of treatment [mean difference (MD), −11.94; 95% confidence interval (CI), −15.45 to −8.43] in a massage group was significantly better than that in route care group. Subgroup analysis suggested that massage with lavender oil also significantly reduced the RLS score after treatment (MD, −14.22; 95% CI, −17.81 to −10.63) and mean difference of RLS score at the beginning and end of treatment (MD, −14.87; 95% CI, −18.29 to −11.45) compared with route care. Meanwhile, massage regime significantly relieved RLS severity compared with route care but did not increase adverse events.

**Conclusion:**

Massage may be a preferred treatment modality for hemodialysis patients with RLS because it effectively reduces RLS symptoms, relieves RLS severity, and does not increase the risk of adverse events. However, future study with a larger sample size is warranted due to the fact that only limited number of eligible studies with small sample size are enrolled.

## Introduction

The prevalence of chronic renal failure (CRF) has considerably increased worldwide, and studies reported a global rate of 10% (1). Patients often face some health challenges after confirming the diagnosis of CRF, and several therapeutic and medical interventions are consequently required (2). Hemodialysis (HD) is one alternative form of the most common renal replacement therapies (RRT) for the treatment of CRF (3). Unfortunately, although HD increased the life expectancy in this patient (4), it was also associated with a series of ailments (5–7), such as pain, constipation, muscle cramps, and restless legs syndrome (RLS) (8, 9).

Restless leg syndrome, also known as Willis-Ekbom disease, is a common neurological and sensorimotor disease (10) which mostly appear or deteriorate during periods of rest or inactivity (11). CRF patients receiving HD treatment commonly suffered from RLS because each HD treatment session required longer inactivity time (12). Hence, it was also named uremic RLS. Studies reported that the incidence of RLS is between 6.6 and 70% among patients undergoing HD (13–15). In addition, RLS was associated with increased severity of fatigue (16), increased quality of sleep disorder (17, 18), and poor quality of life (QoL) (7, 19). It is noted that, currently, RLS has been a top research priority for patients with CRF (18). Meanwhile, studies also indicated that RLS may be associated with increased risk of morbidity and mortality (19–21).

A variety of treatment regimens can be chosen to initially relieve symptoms of patients currently with RLS (14). Among available regimes, pharmacological treatments are mainly selected for cases with severe RLS (22), which, at times, lead to serious complications (23). Consequently, non-pharmacological treatments have been increasingly reported to be useful for the treatment of the syndrome and have fewer unpleasant adverse events (24). Among existing non-pharmacological treatments, massage therapy is frequently prescribed for the management of several complications associated with diseases or treatments (24), and massage with or without adjuvant plant or herbal essences has been selected for the treatment of RLS in recent years (25).

Currently, numerous clinical trials (26–30) have been performed to investigate the therapeutic efficacy of massage among patients with RLS. However, the results of these studies were not relatively reliable due to extremely insufficient sample size. We therefore performed this meta-analysis to further determine whether massage should be preferentially chosen for the management of RLS.

## Materials and Methods

### Study Design

This is a meta-analysis of published studies. As a result, we did not apply for ethical approval and informed consent as we calculated all pooled results at the basis of published data. Moreover, our team performed the current meta-analysis under the methodological framework recommended by the Cochrane Collaboration network (31), and all pooled results were reported according to the Preferred Reporting Items for Systematic Reviews and Meta-Analysis (PRISMA) checklist (32).

### Data Resources

We electronically searched PubMed database, EMBASE database (*via* OVID), and the Cochrane Library to identify all potentially eligible randomized controlled trials (RCTs) published before August 31, 2021. The following essential words were used to construct the search strategy by introducing the principle of combining medical subject heading (MeSH) and full-text word: hemodialysis, restless leg syndrome, and massage. Moreover, we hand-checked reference lists of eligible studies to add additional studies (33). We did not impose language restrictions to limit search results. Search results were double-checked by two independent reviewers, and any disagreements were resolved through consulting with a third reviewer. Finally, we summarized search strategies for all target databases in Supplementary Table 1.

### Selection Criteria

#### Inclusion Criteria

According to the aim designed in the current meta-analysis, we developed our selection criteria based on the PICOS acronym (34) as follows: (a) patients (P): adult hemodialysis patients were diagnosed with RLS based on the criteria developed and approved by the International RLS Study Group (IRLSSG) (35); (b) intervention (I): patients in intervention group were assigned to receive massage regime regardless of adjuvant regimes; (c) comparison (C): patients in the control group were assigned to receive route care; (d) outcomes (O): eligible studies evaluated clinical efficacy of massage regime compared to route care by using calculating RLS scores after treatment and mean difference of RLS scores at the beginning and end of treatment, and meanwhile evaluated safe of regimes through recording adverse events; and (e) study design (S): we only considered RCT to meet our inclusion criteria.

#### Exclusion Criteria

After designing the inclusion criteria, we also developed the following exclusion criteria: (a) patient population of the studies were confirmed to have idiopathic RLS, were consuming medications to manage RLS signs or medications that worsen signs, infection, wound or serious complication in feet, and peripheral neuropathy or vascular problems in lower limbs; (b) ineligible study design such as observational studies, case reports or series, narrative literature reviews, and experimental studies; (c) repeated studies with insufficient data or poor quality; and (d) conference abstract without adequate information.

### Selection of Study

Our team designed the following three steps to select eligible studies: (a) we firstly imported all captured records into EndNote software, and then removed duplicate records. Then, (b) we initially evaluated eligibility of all unique records through screening title and abstract before (c) we retrieved full-texts of all unique records remained from the second step for finally checking eligibility. We resolved any disagreements through consulting a third reviewer.

### Data Extraction

Two independent reviewers were assigned to extract the following data from each eligible study: the name and country of the first author, publication year, sample size, the size of male patients, mean age, details of massage regimes, outcomes, and details of risk of bias. Moreover, we contacted the corresponding author to gain additional data. We calculated the change values and corresponding SD (standard deviation) using the value with SD at baseline and different checkpoints based on a correlation coefficient of 0.5 (36). Any disagreements during this stage were settled by consulting a third reviewer.

### Outcomes of Interest

In this meta-analysis, we investigated RLS score after treatment, mean difference of RLS score before and after treatment, the severity of RLS, and safety of massage. Briefly, severity of RLS was evaluated by using the Restless Legs Syndrome Rating Scale is a self-rating scale, which was originally developed in English to evaluate the severity of RLS (37). In this instrument, ten questions were scored at between zero and four, and a higher overall score indicated a greater RLS severity (38). Safety was evaluated by individual study through recording the incidence of adverse effects.

### Assessment of Risk of Bias

Two independent reviewers assessed the risk of bias of each eligible study using the Cochrane collaboration tool for assessing risk of bias from the following seven items (39): (a) random sequence generation, (b) allocation concealment, (c) blinding of participants and personnel, (d) blinding of outcome assessment, (e) incomplete outcome data, (f) selective reporting, and (g) other sources of bias. Depending on the matching level between actual information and assessment criteria, each of these items was labeled with low, unclear, and high risk. Any disagreements at this stage were settled by consulting a third reviewer.

### Statistical Analysis

#### Heterogeneity Examination

Before performing statistical analysis, we examined heterogeneity across eligible studies. We used the Chi square test (Cochrane Q statistic) to qualitatively assess presence of heterogeneity or not. Then, we utilized I^2^ statistic to quantify the level of heterogeneity (40). It must be noted that we selected a random-effects model to calculate estimates as substantial variations across studies should be acknowledged in the real world.

#### Effect Measures

We used mean difference (MD) and 95% confidence interval (CI) to express the pooled result of continuous variable (clinical efficacy), and used risk ratio (RR) with 95% CI to express the results of dichotomous variables (severity of RLS and adverse events). We conducted the statistical analysis using Review Manager 5.3 (The Nordic Cochrane Centre, the Cochrane Collaboration, Copenhagen).

#### Subgroup Analysis

Considering the fact that lavender oil has been frequently used during massage as an aromatherapy regimen, we therefore separately investigated the therapeutic value of massage with lavender oil for the treatment of RLS among hemodialysis patients by using subgroup analysis technique.

#### Publication Bias Examination

We did not generate funnel plot to examine publication bias as the number of eligible studies was not more than 10 (41).

## Results

### Identification of Studies

**FIGURE 1:**
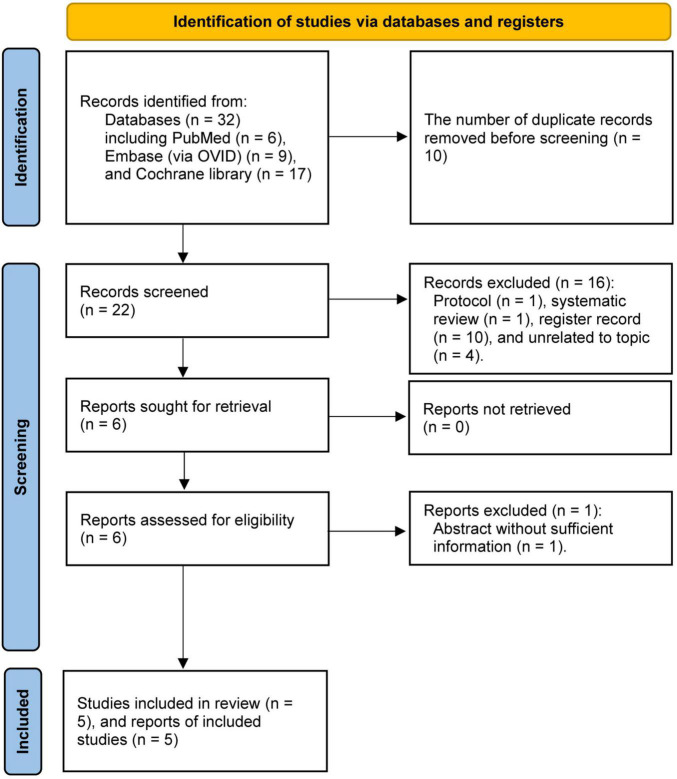
The Preferred Reporting Items for Systematic Review and Meta-analyses (PRISMA) flow chart of capturing and selecting study.

A total of 32 potentially eligible records were identified through an electronic search in three targeted databases including PubMed, EMBASE (*via* OVID), and the Cochrane library. After removing 10 duplicate records by using EndNote software, 22 records were retained for eligibility assessment through screening the title and abstract. Then, we retrieved the full-text of six studies to further evaluate eligibility after removing 16 ineligible studies. Finally, a total of five studies (26–30) were judged to be eligible for our selection criteria after excluding one study due to it being a conference abstract without sufficient information. We summarized the process of searching for and selecting eligible studies in Figure 1.

### Characteristics of Eligible Studies

All enrolled studies were performed in Iran. The publication year of all eligible studies were at between 2005 and 2021. The sample size of individual study ranged from 55 to 105 with a total sample size of 369. Three studies (26, 27, 29) designed a group in which patients were assigned to receive massage with lavender oil, and another two studies assigned patients to only receive reflexology (30) or massage with olive oil (28). The treatment duration was 3 weeks in three studies (26, 29, 30) and 4 weeks in two studies (27, 28). All studies evaluated the clinical efficacy of massage intervention on RLS based on the Restless Leg Syndrome Scores, and only one study (28) evaluated safety through recording adverse events. We summarized the characteristics of all eligible studies in Table 1.

**TABLE 1:** Characteristics of included studies (n = 5).

References	Country	Sample size (male)	Mean age, years	Duration of HD	Details of massage intervention	Treatment duration	Outcomes
Hashemi et al. (26)	Iran	29 (11) vs 30 (17)	57.5 vs 56.1	3.78 vs 3.82	massage with 1.5% lavender oil, twice a week, 10 minutes per session, performed 1 hour after starting the dialysis session.	3 weeks	RLSS
Shahgholian et al. (30)	Iran	30 vs 30	55.45	n.r.	reflexology, three times a week for 30–40 min	4 weeks	RLSS, SRLS
Nasiri et al. (28)	Iran	27 (12) vs 28 (16)	50.6 vs 53.7	n.r.	massage with 10 mL olive oil, twice a week with the total intervention time of 60 min, performed at least 1 h after starting the dialysis session	3 weeks	RISS, SRLS, AEs
Mirbagher Ajorpaz et al. (27)	Iran	60 (27) vs 30 (17)	56.9 vs 56.1	3.67 vs 3.82	massage with 10–15 mL 1.5% lavender and 2% glycerin oil, three times a week for 45 min, performed 1 h after starting the dialysis session	4 weeks	RLSS
Oshvandi et al. (29)	Iran	70 vs 35	51.87	n.r.	massage with 1.5% essential oil of lavender and sweet orange, three times a week, each time for half an hour	3 weeks	RLSS

*HD, hemodialysis; RLSS, Restless Leg Syndrome Scores; SRLS, severity of RLS; AEs, adverse events; n.r., not reported.*

### Risk of Bias

**FIGURE 2:**
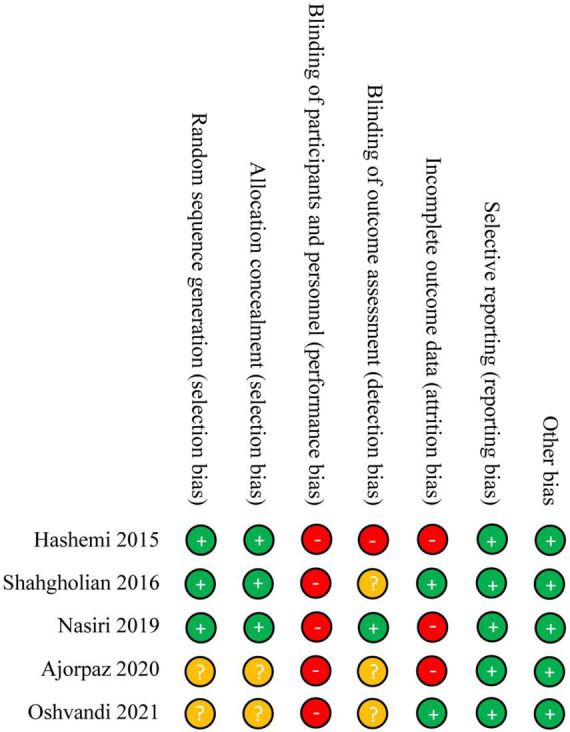
Risk of bias summary of each eligible study.

Among enrolled five studies, three studies (26, 28, 30) appropriately generated random sequence and conducted allocation concealment, four studies (26–29) blinded investigators, one study (28) blinded the outcome assessor, two studies (29, 30) obtained data from all participants who were randomly assigned into each group, all studies reported anticipated outcomes, and all studies did not have risk in other bias sources. However, performance bias was labeled with high risk because patients could not be blinded due to absence of sham massage. The overall quality was considered to be of low level. Details of risk of bias of each study were summarized in Figure 2.

### Meta-Analysis of RLS Score After Treatment

**FIGURE 3:**
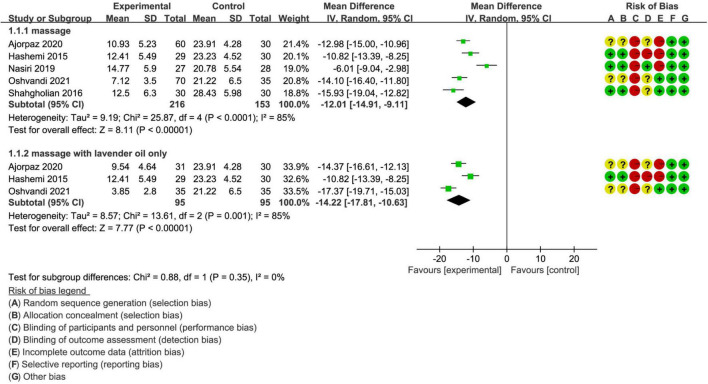
Meta-analysis of International Restless Leg Syndrome Scores (RLSS) after treatment.

All studies reported RLS score after treatment. Summarized result suggested that massage intervention significantly reduced the RLS score compared with route care (MD, −12.01; 95% CI, −14.91 to −9.11; *p* < 0.001; Figure 3), with low to moderate evidence.

Among enrolled 5 studies, three studies (26, 27, 29) assigned patients to separately receive massage with lavender oil, and pooled result suggested that patients were found to have significantly lower RLS scores after received massage with lavender oil compared with those patients received route care (MD, −14.22; 95% CI, −17.81 to −10.63; *p* < 0.001; Figure 3), with low to moderate evidence.

### Meta-Analysis of Mean Difference of RLS Scores

**FIGURE 4:**
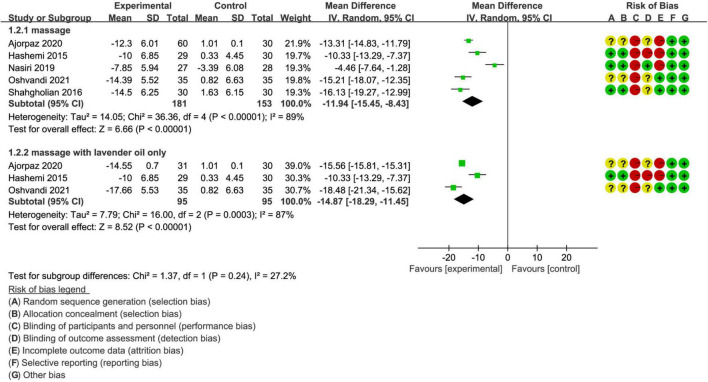
Meta-analysis of mean difference of RLSS at the beginning and end of treatment.

We also calculated the mean difference of RLS score at the beginning and end of treatment to evaluate the efficacy magnitude of massage intervention. Meta-analysis of massage intervention suggested that patients in massage group achieved significantly higher mean differences of RLS scores compared with patients in the route care group (MD, −11.94; 95% CI, −15.45 to −8.43; *p* < 0.001; Figure 4), with low to moderate evidence. Meanwhile, separate analysis of massage with lavender oil also suggested a significantly higher mean difference of RLS scores compared to route care (MD, −14.87; 95% CI, −18.29 to −11.45; *p* < 0.001; Figure 4), with low to moderate evidence.

### Meta-Analysis of Severity of RLS

**FIGURE 5:**
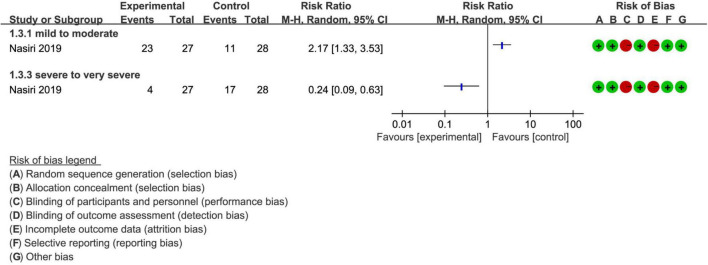
The incidence of different levels of severity after treatment.

Among 5 enrolled studies, only two (28, 30) evaluated improvement in severity of RLS after treatment. However, only one study (28) reported the specific number of different levels of severity, and pooled results suggested that more patients in the massage intervention group were at a mild to moderate level (RR, 2.17; 95% CI, 1.33 to 3.53; Figure 5). Despite this, more patients were still at severe to very severe level (RR, 0.24; 95% CI, 0.09 to 0.63; Figure 5), with low evidence. Meanwhile, Shahgholian et al. also found that the severity of RLS significantly improved immediately after intervention compared with those patients received route care (*p* < 0.001) (30).

### Qualitative Summary of Safety

Among 5 enrolled studies, only one (28) evaluated the safety of massage interventions by recording adverse events after intervention. It is noted that none of the patients experienced any adverse effects connected with the intervention, with low moderate evidence.

## Discussion

As a prevalent neurological disorder that occurs during rest and inactivity, RLS has been reported at a high incidence among CRF parties receiving HD treatment (15). It is noted that RLS can be associated with sleep disturbance and poor quality of life (42) and that non-pharmacological treatments play an important role in managing RLS as many pharmacological treatments had been found to be associated with several adverse events, such as nausea and exacerbation of symptoms (43, 44). As one of the most common non-pharmacological treatments, massage therapy has also been recently used for the management of RLS (24). As far as we know, this is the first attempt to assess the efficacy and safety of massage intervention on RLS symptoms and severity among patients with HD with RLS by using meta-analysis. In this study, we find that massage with or without adjuvant plant or herbal products reduce RLS symptoms and relieve RLS severity but does not increase the incidence of adverse events.

Although the pathophysiology of RLS is less understood (23), abnormal dopamine nervous system pathways is considered to play a critical role in causing such a syndrome. This is because of how it can be relieved by taking a low dosage of levodopa (18). Massage therapy has a potential of stimulating the nervous system and transferring sensory stimuli to the brain by accelerating secretion of dopamine (30, 45). In our meta-analysis, it was seen that RLS symptoms and severity decreased in all enrolled studies (26–30). Meanwhile, our pooled results also consistently indicated that massage intervention effectively reduced the RLS score, with a significantly higher efficacy magnitude compared with route care. Meanwhile, these therapies are recommended due to their minimal adverse effects. Our meta-analysis only enrolled one study which reported the incidence of adverse events after intervention, and no patient was found to have any adverse events (28).

Moreover, in the current meta-analysis, we also separately investigated the therapeutic efficacy of massage with lavender oil for the management of RLS. Lavender essences are known to have several advantages, such as analgesic and anti-depressant effects (46). Studies also found that lavender essences were also helpful in accelerating wound healing and promoting blood circulation (47). Additionally, lavender essences were shown to enhance immunity and relieve muscle aches (27). Our pooled results also suggested that massage with lavender oil effectively improves RLS symptoms and decreases RLS severity because of significantly lower reported RLS scores after intervention, which was consistent with results of three enrolled studies (26, 27, 29).

Although our meta-analysis generates more reliable and robust findings through accumulating more sample size for increasing the statistical power, some limitations must be further interpreted. First, although we enrolled five eligible studies for data analysis, the sample group of individual study is small. Therefore, their results do not constitute sufficient evidence. Second, treatment duration varied among included studies. In addition, we cannot perform subgroup analysis to investigate the impact of different treatment durations on clinical outcomes due to limited number of eligible studies. Third, all eligible studies were performed in Iran. Thus, our findings should be cautiously interpreted in other clinical and cultural settings. Hence, we suggest exploring the efficacy and safety of massage for the treatment of RLS in other settings. Fourth, the formal protocol of this meta-analysis was not registered in a public website. However, we strictly performed it according to the recommendations proposed by the Cochrane Collaboration and reported the results in accordance with PRISMA statement. Fifth, routine interventions of all eligible studies were not clearly described. Therefore, variations of controls may introduce bias. Sixth, although eligible studies used the Restless Legs Syndrome Rating Scale to evaluate the RLS severity, minimally important difference (MID) of this scale was not available, possibly decreasing the accuracy of the measurement of RLS severity. Seventh, we conducted subgroup analysis based on massage protocols to determine whether statistical heterogeneity could be decreased or eliminated. However, substantial heterogeneity was kept for subgroup analyses. Therefore, we must acknowledge that our findings may be negatively influenced by other potential bias sources.

## Conclusion

In the current meta-analysis of 5 RCTs, we found that massage intervention utilized for the treatment of RLS effectively reduces RLS symptoms, relieves RLS severity, and does not increase the risk of adverse events. However, all studies we have enrolled are RCTs with small sample size. Therefore, we suggested to further establish our findings through performing future studies with larger sample size.

## Data Availability Statement

The original contributions presented in the study are included in the article/Supplementary Material, further inquiries can be directed to the corresponding author.

## Author Contributions

All authors listed have made a substantial, direct, and intellectual contribution to the work, and approved it for publication.

## Conflict of Interest

The authors declare that the research was conducted in the absence of any commercial or financial relationships that could be construed as a potential conflict of interest.

## Publisher’s Note

All claims expressed in this article are solely those of the authors and do not necessarily represent those of their affiliated organizations, or those of the publisher, the editors and the reviewers. Any product that may be evaluated in this article, or claim that may be made by its manufacturer, is not guaranteed or endorsed by the publisher.
