# Supplementary material for: Clinical Efficacy and Safety of Massage for the Treatment of Restless Leg Syndrome in Hemodialysis Patients: A Meta-Analysis of 5 Randomized Controlled Trials
Source: Front Psychiatry. 2022 Apr 11;13:843263. doi: 10.3389/fpsyt.2022.843263 (PMC9035587; doi:10.3389/fpsyt.2022.843263)
Supplement: Supplementary file 1 [file Data_Sheet_1.pdf]

**Table S1. Search strategy of target databases**

**Search string of PubMed**

| ID | Query |
|---|---|
| 10 | ((("Massage"[Mesh]) OR (((Massage[Title/Abstract]) OR (zone therapy[Title/Abstract])) OR (zone therapies[Title/Abstract]))) AND (((("Restless Legs Syndrome"[Mesh]) OR "Restless legs syndrome 2" [Supplementary Concept]) OR "Restless legs syndrome 1" [Supplementary Concept]) OR (((((((((Restless Legs Syndrome[Title/Abstract]) OR (restless legs[Title/Abstract])) OR (willis ekbom disease[Title/Abstract])) OR (willis ekbom syndrome[Title/Abstract])) OR (willis ekbom disease[Title/Abstract])) OR (willis ekbom syndrome[Title/Abstract])) OR (wittmaack ekbom syndrome[Title/Abstract])) OR (wittmaack ekbom syndrome[Title/Abstract])) OR (restless leg syndrome[Title/Abstract])))) AND (("Renal Dialysis"[Mesh]) OR ((((((((((((Renal Dialysis[Title/Abstract]) OR (renal dialyses[Title/Abstract])) OR (Hemodialysis[Title/Abstract])) OR (Hemodialyses[Title/Abstract])) OR (extracorporeal dialyses[Title/Abstract])) OR (extracorporeal dialysis[Title/Abstract])) OR (acetate free biofiltration[Title/Abstract])) OR (acetate free biofiltration[Title/Abstract])) OR (Hemodiafiltration[Title/Abstract])) OR (peritoneal dialyses[Title/Abstract])) OR (peritoneal dialysis[Title/Abstract])) OR (CAPD[Title/Abstract]))) |

| | |
|---|---|
| 9 | ("Renal Dialysis"[Mesh]) OR ((((((((((((Renal Dialysis[Title/Abstract]) OR (renal dialyses[Title/Abstract])) OR (Hemodialysis[Title/Abstract])) OR (Hemodialyses[Title/Abstract])) OR (extracorporeal dialyses[Title/Abstract])) OR (extracorporeal dialysis[Title/Abstract])) OR (acetate free biofiltration[Title/Abstract])) OR (acetate free biofiltration[Title/Abstract])) OR (Hemodiafiltration[Title/Abstract])) OR (peritoneal dialyses[Title/Abstract])) OR (peritoneal dialysis[Title/Abstract])) OR (CAPD[Title/Abstract])) |
| 8 | (((((((((((Renal Dialysis[Title/Abstract]) OR (renal dialyses[Title/Abstract])) OR (Hemodialysis[Title/Abstract])) OR (Hemodialyses[Title/Abstract])) OR (extracorporeal dialyses[Title/Abstract])) OR (extracorporeal dialysis[Title/Abstract])) OR (acetate free biofiltration[Title/Abstract])) OR (acetate free biofiltration[Title/Abstract])) OR (Hemodiafiltration[Title/Abstract])) OR (peritoneal dialyses[Title/Abstract])) OR (peritoneal dialysis[Title/Abstract])) OR (CAPD[Title/Abstract]) |
| 7 | "Renal Dialysis"[Mesh] |
| 6 | ((("Restless Legs Syndrome"[Mesh]) OR "Restless legs syndrome 2" [Supplementary Concept]) OR "Restless legs syndrome 1" [Supplementary Concept]) OR (((((((((Restless Legs Syndrome[Title/Abstract]) OR (restless legs[Title/Abstract])) OR (willis ekbom disease[Title/Abstract])) OR (willis ekbom syndrome[Title/Abstract])) OR (willis ekbom disease[Title/Abstract])) OR (willis ekbom syndrome[Title/Abstract])) OR (wittmaack ekbom syndrome[Title/Abstract])) OR (wittmaack ekbom syndrome[Title/Abstract])) OR (restless leg syndrome[Title/Abstract])) |

| | |
|---|---|
| 5 | ((((((((Restless Legs Syndrome[Title/Abstract]) OR (restless legs[Title/Abstract])) OR (willis ekbom disease[Title/Abstract])) OR (willis ekbom syndrome[Title/Abstract])) OR (willis ekbom disease[Title/Abstract])) OR (willis ekbom syndrome[Title/Abstract])) OR (wittmaack ekbom syndrome[Title/Abstract])) OR (wittmaack ekbom syndrome[Title/Abstract])) OR (restless leg syndrome[Title/Abstract]) |
| 4 | (("Restless Legs Syndrome"[Mesh]) OR "Restless legs syndrome 2" [Supplementary Concept]) OR "Restless legs syndrome 1" [Supplementary Concept] |
| 3 | ("Massage"[Mesh]) OR (((Massage[Title/Abstract]) OR (zone therapy[Title/Abstract])) OR (zone therapies[Title/Abstract])) |
| 2 | ((Massage[Title/Abstract]) OR (zone therapy[Title/Abstract])) OR (zone therapies[Title/Abstract]) |
| 1 | "Massage"[Mesh] |

**Search string of Embase (via OVID)**

| ID | Search |
|---|---|
| 1 | (zone therapy or zone therapies or Massage).af. |
| 2 | Massage.sh. |
| 3 | 1 or 2 |
| 4 | (Restless Legs Syndrome or restless legs or willis ekbom disease or willis ekbom syndrome or willis ekbom disease or willis ekbom syndrome or wittmaack ekbom syndrome or wittmaack ekbom syndrome or restless leg syndrome).af. |
| 5 | Restless Legs Syndrome.sh. |
| 6 | 4 or 5 |
| 7 | (Renal Dialysis or renal dialyses or Hemodialysis or Hemodialyses or extracorporeal dialyses or extracorporeal dialysis or acetate free biofiltration or acetate free biofiltration or Hemodiafiltration or peritoneal dialyses or peritoneal dialysis or CAPD).af. |
| 8 | Renal Dialysis.sh. |
| 9 | 7 or 8 |
| 10 | 3 and 6 and 9 |
| 11 | limit 10 to embase |

**Search string of the Cochrane library**

ID Search

#1 (Massage):ti,ab,kw OR (zone therapy):ti,ab,kw OR (zone therapies):ti,ab,kw

#2 MeSH descriptor: [Massage] explode all trees

#3 #1 or #2

#4 (Restless Legs Syndrome):ti,ab,kw OR (restless legs):ti,ab,kw OR (willis ekbom disease):ti,ab,kw OR (willis ekbom syndrome):ti,ab,kw OR (willis ekbom disease):ti,ab,kw

#5 (willis ekbom syndrome):ti,ab,kw OR (wittmaack ekbom syndrome):ti,ab,kw OR (wittmaack ekbom syndrome):ti,ab,kw OR (restless leg syndrome):ti,ab,kw

#6 MeSH descriptor: [Restless Legs Syndrome] explode all trees

#7 #4 or #5 or #6

#8 (Renal Dialysis):ti,ab,kw OR (renal dialyses):ti,ab,kw OR (Hemodialysis):ti,ab,kw OR (Hemodialyses):ti,ab,kw OR (extracorporeal dialyses):ti,ab,kw

#9 (extracorporeal dialysis):ti,ab,kw OR (acetate free biofiltration):ti,ab,kw OR (acetate free biofiltration):ti,ab,kw OR (Hemodiafiltration):ti,ab,kw OR (peritoneal dialyses):ti,ab,kw

#10 (peritoneal dialysis):ti,ab,kw OR (CAPD):ti,ab,kw

#11 MeSH descriptor: [Renal Dialysis] explode all trees

#12 #8 or #9 or #10 or #11

#13 #3 and #7 and #12
